# Three-Dimensional Conformal Radiotherapy Combined with Gemcitabine and Docetaxel in the Treatment of Advanced Bladder Cancer and Its Effects on Inflammatory Factors and Immune Function

**DOI:** 10.1155/2022/9347218

**Published:** 2022-04-14

**Authors:** Jianwei Li, Min Liu, Pengyu Sun, Wanli Zhao, Miaomiao Song, Yang Liu

**Affiliations:** 2nd Department of Urology, Cangzhou Central Hospital, Cangzhou, China

## Abstract

**Objective:**

To assess the efficacy of three-dimensional conformal radiotherapy (3D-CRT) combined with GT chemotherapy (gemcitabine+docetaxel) in the treatment of advanced bladder cancer and its influence on inflammatory factors and immune function.

**Methods:**

A total of 42 elderly patients with advanced bladder cancer who were admitted to our hospital from January 2019 to January 2020 were included and assigned to the GT group (21 cases) receiving GT chemotherapy and combination group (21 cases) given 3D-CRT combined with GT chemotherapy. The clinical efficacy, immune function, inflammatory factors, tumor markers, urinary angiogenesis molecules before and after treatment, 1-year survival rate, 2-year survival rate, and incidence of adverse reactions of the two groups were compared. SPSS 22.0 statistical software was used for data processing and analysis.

**Results:**

The combination group had 5 cases of CR, 12 cases of PR, 3 cases of SD, and 1 case of PD, with an ORR of 80.95% (17/21), which was remarkably higher than the ORR of 57.14% (12/21) in the GT group which had 3 cases of CR, 9 cases of PR, 5 cases of SD, and 4 cases of PD (*P* < 0.05). The 1-year survival rate of the combination group was 76.19% (16/21), and the 2-year survival rate was 47.62% (10/21), which were higher than the 1-year survival rate of 47.62% (10/21) and 2-year survival rate of 19.05% (4/21) in the GT group (*P* < 0.05). The two groups presented similar results in terms of adverse reactions rate (*P* > 0.05). After treatment, the combination group obtained significantly lower levels of urinary bladder cancer antigen (UBC), nuclear matrix protein-22 (NMP-22), vascular endothelial growth factor (VEGF), and basic fibroblast growth factor (bFGF) than the GT group (*P* < 0.05). The CD3+, CD4+, and CD4+/CD8+ levels of the two groups of patients were lower than those before treatment (*P* < 0.05), but no statistical difference was observed between the two groups after treatment (*P* > 0.05). The levels of interleukin-6 (IL-6) and interferon-*γ* (IFN-*γ*) of the two groups witnessed a decline after treatment, with lower results in the combination group as compared to the control group (*P* < 0.05). Before treatment, no significant difference in the Generic Quality of Life Inventory-74 (GQOLI-74) score between the two groups was found (*P* > 0.05). After treatment, the combination group had higher GQOLI-74 scores than the GT group (*P* < 0.05).

**Conclusion:**

3D-CRT combined with GT chemotherapy yields a significant effect on the treatment of elderly advanced bladder cancer by effectively protecting immune function, mitigating inflammation, inhibiting tumor marker levels and the expression of angiogenic molecules, and improving patients' survival.

## 1. Introduction

Bladder cancer is a malignant tumor on the human bladder mucosa which is commonly seen in clinical practices. It ranks first in the incidence of genitourinary system tumors in China [1]. To our knowledge, gemcitabine is a pyrimidine antitumor drug, and docetaxel is a phase M cycle-specific drug. Studies have shown that gemcitabine combined with docetaxel sequential maintenance intravesical instillation in the treatment of nonmuscle invasive bladder cancer can effectively improve the 1-year and 2-year disease-free survival rates of patients [2]. Nevertheless, some studies have found that a single chemotherapy treatment might be less than satisfactory; the worse yet is that advanced bladder cancer tumors are poorly differentiated and have a high degree of malignancy, rendering the treatment more challenging [3]. Three-dimensional conformal radiotherapy (3D-CRT) is a high-precision radiotherapy, by which three-dimensional tumor structure can be reconstructed via CT. The distribution shape of the dose area is consistent with the shape of the target area three-dimensionally through different directions and different irradiation fields; the conformal plumbic blocks that are identical to the shape of the lesion, so as to reduce the dose to the normal tissue around the lesion. It has been reported that 3D-CRT combined with chemotherapy yields a promising therapeutic effect in malignant bladder tumors, with a good safety profile, and merits promotion [4]. Li [5] argued that compared with radiotherapy alone, 3D-CRT combined with gemcitabine exhibited superior performance in the total clinical response rate of elderly patients with advanced bladder cancer, with a good safety profile. Nevertheless, the study on the treatment of advanced bladder cancer with 3D-CRT combined with gemcitabine and docetaxel has yet been scarcely reported. Encouragingly, the combination of radiotherapy and chemotherapy has captured extensive attention in the treatment of bladder cancer in recent years. The present study was designed to further optimize the treatment schedule for patients with advanced bladder cancer and to explore its impact on inflammatory factors and immune function.

The report is as follows.

## 2. Materials and Methods

### 2.1. General Information

A total of 42 elderly patients with advanced bladder cancer who were treated from January 2019 to January 2020 were included and randomized (1 : 1) into the GT group (GT chemotherapy) and the combined group (GT chemotherapy plus 3D-CRT) via a random number table method. There were 15 males and 6 females in the combination group. The average age was 73.05 ± 5.53 years, and the diameter of the tumor was 4.8~7.2 cm, with an average of 6.17 ± 0.37 cm; in the TNM stage, 12 cases were in stage III and 9 cases were in stage IV. There were 14 males and 7 females in the GT group. The average age was 73.22 ± 5.31 years, and the diameter of the tumor was 5.1~7.8 cm, with an average of 6.36 ± 0.39 cm; in the TNM stage, 13 cases were in stage III and 8 cases were in stage IV. The two groups presented no great disparity in terms of general information (*P* > 0.05). See Table 1.

### 2.2. Inclusion Criteria [6]

Advanced bladder cancer is confirmed by pathology; the pathological stage was IIIb~IV; age ≥ 60; estimated survival time > 3 months; the patient had a clear lesion; none received other antitumor therapy before enrollment. Patients and their families had a clear understanding of chemotherapy. Exclusion criteria were as follows: combined with other malignant tumors; combined with liver and kidney dysfunction; with chemotherapy contraindications; and complicated with chronic diseases such as diabetes, hypertension, and heart disease.

### 2.3. Treatment Methods

The GT group received GT chemotherapy. Before chemotherapy, palonosetron and diphenhydramine were applied to prevent gastrointestinal reactions, and dexamethasone was used to prevent gastrointestinal allergies. On the first and eighth day, gemcitabine (registration number h20160225) 1000 mg/m^2^ was injected intravenously. On the second day, docetaxel (registration number h20150113) was injected intravenously at a dose of 70 mg/m^2^. A course of treatment included four weeks, and two courses of treatment were given, with the intermittent period of three weeks.

The combination group was given 3D-CRT combined with chemotherapy. 3D-CRT: a 16-slice spiral CT scan was used for positioning, and the tumor volume (GTV), namely, the range of the primary lesion and metastatic lymph nodes, was recorded according to the CT positioning image. The clinical target volume (CTV) was 0.5~1.0 cm, and the planning target volume (PTV) was 1 cm. The MM50 conformal intensity-modulated radiotherapy apparatus from Scanditronix of Sweden was used for the radiation treatment, with 5~7 noncoplanar asymmetric radiation fields. The 90% isodose curve method centered on PTV was used to design the irradiation dose which was optimized through the isodose curve and the irradiation dose histogram. The bladder was emptied 1 hour before radiotherapy, and 40 mL of normal saline was injected into the bladder to ensure a full bladder. The bladder was irradiated with 6MV-X rays, 2 Gy/time, 1 time/d, 5 times/week, 4 weeks as a course of treatment. Two courses of treatment were given, with the intermittent period of three weeks.

### 2.4. Observational Indicators

1. The treatment efficacy of the two groups of patients: one month after radiotherapy, lesion examination, chest X-ray, and multiparameter magnetic resonance imaging (mpMRI) were performed, and the patients' treatment effect was evaluated according to the evaluation criteria of solid tumors. The criteria include complete response (CR), partial response (PR), stable disease (SD), and progressive disease (PD). The objective response rate (ORR) = (CR + PR)/total number of cases × 100%
2. The immune function of the two groups before treatment and 3 days after treatment was compared. T lymphocyte subsets CD3+, CD4+, and CD4+/CD8+ were detected by flow cytometry
3. The inflammatory factors of the two groups before treatment and 3 days after treatment were compared. Three millimeters of fasting venous blood was collected from all patients, followed by centrifugation to obtain the serum. An enzyme-linked immunosorbent assay was used to determine the levels of interleukin-6 (IL-6) and interferon-*γ* (IFN-*γ*)
4. The levels of tumor markers before and after treatment in the two groups were compared, including urinary bladder cancer antigen (UBC), nuclear matrix protein-22 (NMP-22), and urinary angiogenesis molecules that contain vascular endothelial growth factors (VEGF) and basic fibroblast growth factors (bFGF). Three millimeters of morning fasting urine was collected and centrifuged to remove the sediment, and the levels of UBC, NMP-22, VEGF, and bFGF in the urine were measured with an enzyme-linked immunoassay kit provided by Shanghai Kehua Bioengineering Co., Ltd
5. All patients were followed up for two years, and their survival was recorded by outpatient follow-up or telephone. The 1-year and 2-year survival rates and the incidence of adverse reactions between the two groups were compared
6. The quality of life of the two groups of patients before and after treatment was compared. Generic Quality of Life Inventory-74 (GQOLI-74) was used to evaluate the quality of life of patients. GQOLI-74 includes four dimensions: material function, social function, mental function, and physical function, and consists of 20 factors and 74 items. Each item uses a 5-level (1-5) scoring method, with a minimum score of 4 points

### 2.5. Statistical Methods

SPSS 22.0 statistical software was used for data processing and analysis. Counting data were expressed as mean ± standard deviation, and the comparison between groups was analyzed by the *t*-test. The *χ*^2^ test was used for the comparison of measurement data between groups. *P* < 0.05 is considered statistically significant.

## 3. Results

### 3.1. Comparison of Efficacy

The combination group had 5 cases of CR, 12 cases of PR, 3 cases of SD, and 1 case of PD, with an ORR of 80.95% (17/21), which was remarkably higher than the ORR of 57.14% (12/21) in the GT group which had 3 cases of CR, 9 cases of PR, 5 cases of SD, and 4 cases of PD (*P* < 0.05). See Table 2.

### 3.2. Comparison of Tumor Markers

There was no significant difference in urine UBC and NMP-22 levels between the two groups before treatment (*P* > 0.05). After treatment, lower urine UBC and NMP-22 levels were observed in the combination group than those in the GT group (*P* < 0.05). See Table 3.

### 3.3. Comparison of Angiogenesis Molecules

The two groups showed no significant difference in urine VEGF and bFGF levels before treatment (*P* > 0.05). After treatment, the combination group had lower urine VEGF and bFGF levels in contrast to the GT group (*P* < 0.05). See Table 4.

### 3.4. Comparison of Immune Function

The CD3+, CD4+, and CD4+/CD8+ levels of the two groups of patients were lower than those before treatment (*P* < 0.05), but no statistical difference was observed between the two groups after treatment (*P* > 0.05). See Table 5.

### 3.5. Comparison of Inflammatory Factors

The levels of interleukin-6 (IL-6) and interferon-*γ* (IFN-*γ*) of the two groups witnessed a decline after treatment, with lower results in the combination group as compared to the control group (*P* < 0.05). See Table 6.

### 3.6. Survival Rate Comparison

The 1-year survival rate of the combination group was 76.19% (16/21), and the 2-year survival rate was 47.62% (10/21), which were higher than the 1-year survival rate of 47.62% (10/21) and 2-year survival rate of 19.05% (4/21) in the GT group (*P* < 0.05). See Table 7.

### 3.7. Comparison of the Incidence of Adverse Reactions

The two groups presented similar results in terms of adverse reaction rate (*P* > 0.05). See Table 8.

### 3.8. Comparison of Quality of Life before and after Treatment

Before treatment, no significant difference in the Generic Quality of Life Inventory-74 (GQOLI-74) score between the two groups was found (*P* > 0.05). After treatment, the combination group had higher GQOLI-74 scores than the GT group (*P* < 0.05). See Table 9.

## 4. Discussion

Advanced bladder cancer indicates a terminal stage of the disease [7], in which common symptoms mainly include increased hematuria that leads to blood clots in the bladder, severe dysuria, and urinary tract obstruction [8]. In severe cases, the development of urinary tract infection will result in lower urinary tract irritation, and metastatic symptoms may also occur, such as lymphedema and lower extremity edema, which will eventually result in systemic symptoms such as malnutrition and weight loss. Patients with advanced bladder cancer have lost the optimal surgical opportunity [9], for whom palliative radiotherapy or chemotherapy can only be used to prolong their survival. In light of the poor tolerance of patients with advanced bladder cancer, chemotherapy drugs with fewer side effects were more appreciated. GT chemotherapy combined with gemcitabine and docetaxel is currently a commonly used regimen for patients with advanced bladder cancer [10]. Gemcitabine is a cycle-specific antitumor drug that can effectively inhibit mitosis, while docetaxel can prominently maintain blood vessel stability. The combination of the two drugs contributes to the enhancement of the antitumor effects; however, unsatisfactory efficacy has also been observed in some cases, which underlines the importance of the combination of other protocols to reinforce the antitumor effect [11]. 3D-CRT is currently the mainstream technology of radiotherapy, with the main feature of the designable radiation field according to the shape of the tumor in the CT scan target area, which can effectively improve the accuracy and targeting of the dose distribution, to ensure lower radiation doses to normal tissues, enhance local control rates, and ameliorate the quality of life [12].

Results of this study demonstrated that the objective response rate (ORR), 1-year survival rate, and 2-year survival rate of the combination group were higher than those of the GT group. The two groups presented similar results in terms of adverse reaction rate. This result suggests that the two drugs are mutually reinforcing in terms of efficacy, thereby enhancing the antitumor effect and prolonging the survival time of patients. The level of tumor markers in urine is an important indicator to evaluate the condition of bladder cancer [13]. UBC is a specific tumor marker for patients with bladder cancer, which is mainly derived from bladder cancer cells. NMP-22 is the main component of mitotic protein and is closely related to the proliferation of bladder cancer [14]. In addition, angiogenic molecules are involved in the regulation of angiogenesis in bladder cancer. VEGF can directly induce the proliferation of endothelial cells and promote angiogenesis, and bFGF can induce the proliferation of endothelial cells and fibroblasts and promote the maturation of neovascular structures [15]. After treatment, the combination group obtained significantly lower levels of urinary bladder cancer antigen (UBC), nuclear matrix protein-22 (NMP-22), vascular endothelial growth factor (VEGF), and basic fibroblast growth factor (bFGF) than the GT group. 3D-CRT can substantially kill tumor cells in the primary tumor lesion through targeted irradiation. The mutual promotion of 3D-CRT and chemotherapy reduce residual tumor cells in the whole body, inhibit tumor cell division and proliferation, bring down tumor marker levels, and further control disease progression.

Th1 and Th2 can regulate the body's inflammatory response. IL-6 is derived from Th2 cells and presents abnormal changes in inflammatory responses [16]. IFN-*γ* is secreted by Th1 cells, which can recognize tumor cells and inhibit the proliferation of tumor cells and tumor angiogenesis [17]. Related research pointed out [18] that radiotherapy and chemotherapy can inhibit the immune function of the body, with the most obvious change detected in T lymphocytes, of which the main clinical manifestations are the reduction of CD3+, CD4+, and CD4+/CD8+. In this study, the CD3+, CD4+, and CD4+/CD8+ levels of the two groups of patients were lower than those before treatment, but no statistical difference was observed between the two groups after treatment. The levels of IL-6 and IFN-*γ* of the two groups witnessed a decline after treatment, with lower results in the combination group as compared to the control group, suggesting that the combination of conformal radiotherapy and GT yields little impact on immune function and contributes to abating inflammation.

With the development of medicine, the quality of life of patients with malignant tumors has captured growing clinical attention [19]. A study has found [20] that the quality of life of patients with malignant tumors is affected by factors such as negative emotions, symptoms, social support, age, education level, and disease awareness [21, 22]. Before treatment, no significant difference in the Generic Quality of Life Inventory-74 (GQOLI-74) score between the two groups was found. After treatment, the combination group had higher GQOLI-74 scores than the GT group, suggesting that the combination of conformal radiotherapy and chemotherapy presents no major negative impact on the quality of life of patients with advanced bladder cancer. Moreover, it contributes to relieving symptoms and reducing the occurrence of nausea and vomiting during treatment, thereby improving the patients' quality of life.

In conclusion, 3D-CRT combined with GT chemotherapy yields a significant effect on the treatment of elderly advanced bladder cancer by effectively protecting immune function, mitigating inflammation, inhibiting tumor marker levels and the expression of angiogenic molecules, and improving patients' survival.

## Figures and Tables

**Table 1:** Comparison of general information of the two groups of patients.

Groups	*n* (case)	Gender		Age (year)	Tumor diameter (cm)	TNM staging	
		Male	Female			III	IV
Combination group	21	15	6	73.05 ± 5.53	6.17 ± 0.37	12	9
GT group	21	14	7	73.22 ± 5.31	6.36 ± 0.39	13	8
*t*/*χ*^2^		0.079		0.454	0.351	0.099	
*P*		0.683		0.164	0.122	0.753	

**Table 2:** Comparison of efficacy between the two groups.

Groups	*n*	CR	PR	SD	PD	ORR
Combination group	21	5	12	3	1	17 (80.95%)
GT group	21	3	9	5	4	12 (57.14%)
*χ* ^2^						4.953
*P*						0.025

**Table 3:** Comparison of tumor markers between the two groups.

Groups	*n*	UBC (*μ*g/L)		*t*	*P*	NMP-22 (U/mL)		*t*	*P*
		Before treatment	After treatment			Before treatment	After treatment		
Combination group	21	22.66 ± 3.29	12.41 ± 1.32	12.328	<0.001	18.09 ± 3.28	10.75 ± 1.16	15.145	<0.001
GT group	21	22.13 ± 3.45	15.52 ± 1.53	7.311	<0.001	18.46 ± 3.39	13.62 ± 1.27	8.527	<0.001
*t*		0.219	5.821			0.150	5.414		
*P*		0.615	<0.001			0.821	<0.001		

**Table 4:** Comparison of angiogenesis molecules between the two groups.

Groups	*n*	VEGF (ng/mL)		*t*	*P*	bFGF (ng/mL)		*t*	*P*
		Before treatment	After treatment			Before treatment	After treatment		
Combination group	21	13.23 ± 1.32	4.42 ± 0.6	11.088	<0.001	10.03 ± 1.54	3.21 ± 1.14	14.182	<0.001
GT group	21	13.37 ± 1.55	6.83 ± 0.8	6.301	<0.001	10.27 ± 1.42	5.26 ± 1.20	7.507	<0.001
*T*		0.632	5.129			0.340	5.514		
*P*		0.352	<0.001			0.511	<0.001		

**Table 5:** Comparison of immune function between the two groups.

Groups	*n*	CD3+ (%)				CD4+ (%)				CD4+/CD8+			
		Before treatment	After treatment	*t*	*P*	Before treatment	After treatment	*t*	*P*	Before treatment	After treatment	*t*	*P*
Combination group	21	58.32 ± 6.35	56.54 ± 5.03	3.041	0.005	35.21 ± 4.15	33.13 ± 3.95	4.172	0.003	1.38 ± 0.17	1.25 ± 0.16	6.961	<0.001
GT group	21	58.42 ± 6.36	56.12 ± 5.42	7.226	<0.001	35.17 ± 4.32	33.85 ± 3.12	8.532	<0.001	1.40 ± 0.16	1.22 ± 0.15	4.326	0.002
*T*		0.067	0.841			0.075	0.984			0.095	0.856		
*P*		0.921	0.312			0.872	0.307			0.752	0.415		

**Table 6:** Comparison of inflammatory factors between the two groups.

Groups	*n*	IL-6 (ng/L)		*t*	*P*	IFN-*γ* (ng/L)		*t*	*P*
		Before treatment	After treatment			Before treatment	After treatment		
Combination group	21	5.28 ± 0.68	63.43 ± 7.95	21.125	<0.001	10.03 ± 1.54	6.69 ± 0.86	8.677	<0.001
GT group	21	5.15 ± 0.62	132.75 ± 16.12	26.352	<0.001	10.53 ± 1.35	7.84 ± 0.94	7.494	<0.001
*T*		0.851	18.132			0.761	4.136		
*P*		0.351	<0.001			0.472	<0.001		

**Table 7:** Comparison of survival rates between the two groups.

Groups	*n*	1-year survival rate	2-year survival rate
Combination group	21	16 (76.19%)	10 (47.62%)
GT group	21	10 (47.62%)	4 (19.05%)
*χ* ^2^		4.215	5.821
*P*		0.013	0.009

**Table 8:** Comparison of the incidence of adverse reactions between the two groups of patients.

Groups	*n*	Decreased platelets	Decreased white blood cells	Nausea and vomiting	Liver damage	Total incidence
Combination group	21	1	1	2	1	5 (23.81%)
GT group	21	2	1	3	1	7 (33.33%)
*χ* ^2^						0.953
*P*						0.425

**Table 9:** Comparison of quality of life before and after treatment.

Groups	*n*	GQOLI-74		*t*	*P*
		Before treatment	After treatment		
Combination group	21	191.52 ± 23.12	224.53 ± 33.34	6.815	<0.001
GT group	21	191.81 ± 23.25	206.58 ± 30.31	5.378	<0.001
*T*		0.311	2.152		
*P*		0.702	0.006		

## Data Availability

The datasets used during the present study are available from the corresponding author upon reasonable request.
